# Investigating the Effect of Grinding Time on High-Speed Grinding of Rails by a Passive Grinding Test Machine

**DOI:** 10.3390/mi13122118

**Published:** 2022-11-30

**Authors:** Pengzhan Liu, Wenjun Zou, Jin Peng, Furen Xiao

**Affiliations:** 1 Henan Engineering Lab for Super-Hard Grinding Gomposites, College of Materials Science & Engineering, Henan University of Technology, Zhengzhou 450007, China; 2 Key Lab of Metastable Materials Science & Technology, Hebei Key Lab for Optimizing Metal Product Technology and Performance, College of Materials Science & Engineering, Yanshan University, Qinhuangdao 066004, China

**Keywords:** high-speed rail grinding, passive grinding, grinding time, grinding pass, grinding wheel

## Abstract

High-speed rail grinding is a unique passive grinding maintenance strategy that differs from conventional grinding techniques. Its grinding behavior is dependent on the relative motion between the grinding wheel and rail; hence, it possesses great speed and efficiency. In this study, the effects of the duration of grinding time and the increase in the number of grinding passes on the grinding of high-speed rails were investigated using passive grinding tests with a single grinding time of 10 s and 30 s and grinding passes of once, twice, and three times, respectively. The results show that when the total grinding time was the same, the rail removal, grinding ratio of grinding wheels, rail grinding effect, grinding force, and grinding temperature were better in three passes of 10 s grinding than in one pass of 30 s grinding, indicating that the short-time and multi-pass grinding scheme is not only conducive to improving the grinding efficiency and grinding quality in the high-speed rail grinding but can also extend the service life of the grinding wheels. Moreover, when the single grinding times were 10 s and 30 s, respectively, the grinding removal, grinding efficiency, grinding marks depth, and surface roughness of rail increased with the number of grinding passes, implying that the desired rail grinding objective can be achieved by extending the grinding time via the multi-pass grinding strategy. The results and theoretical analysis of this study will contribute to re-conceptualizing the practical operation of high-speed rail grinding and provide references for the development of the grinding process and grinding technology.

## 1. Introduction

As one of the principal modes of contemporary transportation, railways hold an unrivaled strategic position and significance [1,2]. The construction of high-speed railway networks in China, Japan, and Western Europe has been essentially completed, giving the nearly 200-year-old industry new vigor and vitality [3,4]. This is especially true with the introduction of a large number of high-speed trains traveling at speeds of more than 250 km per hour [5]. With the growth in train operation hours, frequency, and load capacity, however, the damage caused by trains to railways is increasing daily. This has resulted in a substantial increase in the number and generation rate of rail defects [6]. Currently, rail grinding is widely recognized as an advanced and effective technique for rail maintenance in the leading railway nations, as it can eliminate a variety of rail defects, including wave wear, rolling contact fatigue (RFC), and rail wear damage, without disassembling the rail [7]. This accomplishes the purposes of restoring the rail surface, limiting rail surface fatigue, and reshaping the rail profile [8]. There are multiple methods for grinding rails. Active grinding is the prevalent approach for rails, in which the motor rotates the grinding wheels to grind the rail [9]. Active rail grinding is characterized by its sluggish operation speed and substantial rail wear. This is a maintenance-type grinding strategy developed in the early stages of rail maintenance technology to repair rail surface damage. It largely eliminates existing rail surface defects that affect the operational quality of trains by removing a substantial amount of rail [10]. Nevertheless, since the time spent on rail maintenance operations in the rail transportation plan is being reduced year by year with the increase of train operation time, in addition to a large amount of rail grinding that will reduce the service life of rails, the concept of rail maintenance grinding is gradually shifting from maintenance-type grinding with extensive metal removal to preventive grinding operations with short intervals and less metal removal [11,12]. Therefore, the active rail grinding method has become less adapted to the current high-speed rail transportation situation, and a new high-speed rail grinding technology has come into being [13,14].

**Figure 1:**
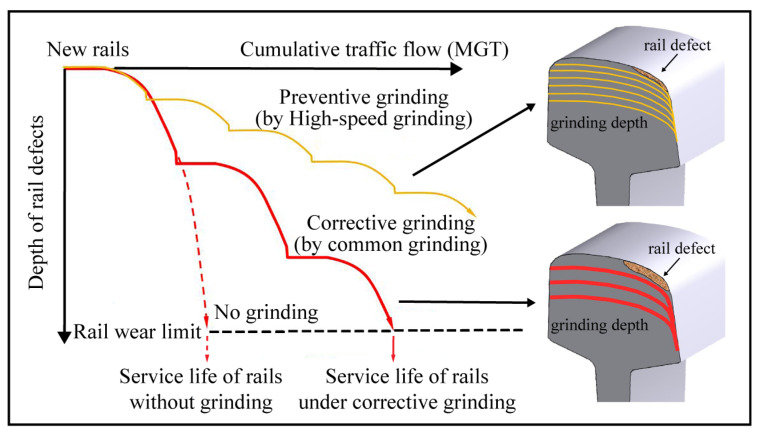
Effect of different grinding technologies on the service life of rails [17,21].

Because there is no active drive for the grinding wheel, rail high-speed grinding is also known as passive grinding [15]. High speed refers to the forward speed of the grinding train, not the linear speed of the grinding wheel. The grinding wheel needs to follow the high-speed grinding train to advance rapidly on the surface of the rail, relying solely on the grinding pressure provided by the passive grinding mechanism and the friction force generated by the relative movement of the grinding wheel and the rail to passively rotate and complete the grinding behavior of rail [16]. The structure design and operation principle of passive high-speed grinding allow the grinding train to execute rail grinding at speeds between 60 and 80 km per hour. In contrast to the common active grinding train at 3 and 15 km per hour, the passive high-speed grinding train does not require a unique “window” to close the railway and does not require the dismantling of any railway signal equipment or rail accessories prior to grinding [17]. Consequently, the adoption of passive high-speed grinding technology in the preventive grinding of rail has unmatched efficiency and cost advantages. For the application of rail high-speed grinding technology, the United Kingdom, Germany, and other nations have spent over a decade demonstrating through practice the unparalleled application value of high-speed grinding technology for rail preventive grinding in the long straight length of rail track [18,19]. According to the data and technical experience acquired by the German railway maintenance, the preventive grinding maintenance of rails every four months using high-speed grinding technology can achieve the same rail maintenance effect as the repairing grinding maintenance by active grinding performed every 4.5 MGT of the total train passing weight [20]. This considerably extends the rails’ service life, as demonstrated in Figure 1 [17,21].

Notably, the rail high-speed grinding maintenance process must sometimes adjust the rail grinding time based on the state of rail surface wear to accomplish the desired effect of regulating rail grinding [22]. There are two conventional methods for controlling the grinding time. One is to increase or decrease the length of the grinding train to change the number of high-speed grinding wheels, thereby altering the time of grinding in a single pass of high-speed grinding; the other is to perform multiple grindings of rails during a maintenance operation, which is also a disguise to alter the time of grinding wheels grinding rails [23]. Since increasing the length of the grinding train will raise the operating costs of rail maintenance and there are upper limits on the length of the grinding train, the passive grinding process of repeated multiple passes in one rail maintenance is generally used to regulate the high-speed grinding time [8].

However, in addition to the significant differences in rail service life, grinding wheel life and rail maintenance effects caused by the difference in total grinding time, the single long-time passive grinding process, and the multiple short-time passive grinding process will produce different results for high-speed rail grinding with the same total grinding time. Yet, there is little relevant research studying the impact of grinding time on railways, and the majority of discussions regarding rail grinding time focus on the prevalent active rail grinding technique. Lin et al. [24] investigated the influence of grinding time and grinding pass of active rail grinding on rail surface temperature and burn behavior. This study found that the surface of the rail samples was burned to varying degrees when the rotating speed of the grinding stone exceeded 2600 r/min and the grinding pressure above 1000 N during the 8 s active rail grinding tests. Moreover, when the linear velocity of the grinding stone and the pressure were held constant, the maximum grinding temperature rose differently as the number of grinding passes increased. This shows that the variation of grinding time affects the surface grinding quality of the rails. Zhang et al. [25] discussed the impact of grinding passes and direction on the behavior of material removal in the rail grinding process. The authors illustrated that the increase in the number of grinding passes increased the wear of the grinding wheel, resulting in a decrease in rail removal, grinding efficiency, and rail surface roughness, by developing a three-dimensional finite element model of active rail grinding. Wu et al. [26] examined the effect of grinding time on the wear characteristics of brazed diamond grinding tools for rail grinding using a custom-built active rail grinding tester. This study revealed that the intermittent grinding strategy of single grinding for 6 s and cumulative grinding for 5 passes resulted in less grinding tool wear and rail surface roughness than the single pass grinding for 30 s. This showed that the short time and multi-pass grinding process extended the service life of the grinding tools and optimized the rail surface morphology after grinding. The aforementioned studies on active rail grinding provide a reference for the setting of grinding time parameters in high-speed rail grinding, but they cannot be used to guide the grinding time in high-speed rail grinding line operations or to illustrate the effect of single long-time passive grinding and multiple short-time passive grinding on high-speed rail grinding results and grinding wheels.

The purpose of this article was to optimize the effect of rail high-speed grinding and extend the service life of high-speed grinding wheels, and a self-designed passive grinding test machine was used to investigate the effect of grinding time and grinding passes on the high-speed grinding of rails. During grinding, the grinding efficiency, service life of the grinding wheel, grinding effect on rails, grinding force, and grinding temperature are discussed. Due to the fact that water, coolant, and other grinding media are undesirable in practical rail grinding operations, dry grinding is the appropriate grinding state. This is because rail maintenance must take into account operating safety and environmental protection. Consequently, the focus of this work is on the outcomes of grinding tests conducted under dry grinding conditions with no further grinding aids employed. This study will contribute to an in-depth understanding of the passive grinding operation in high-speed rail grinding and to the formulation of grinding process specifications.

## 2. Experiment

### 2.1. Grinding Testing Machine

**Figure 2:**
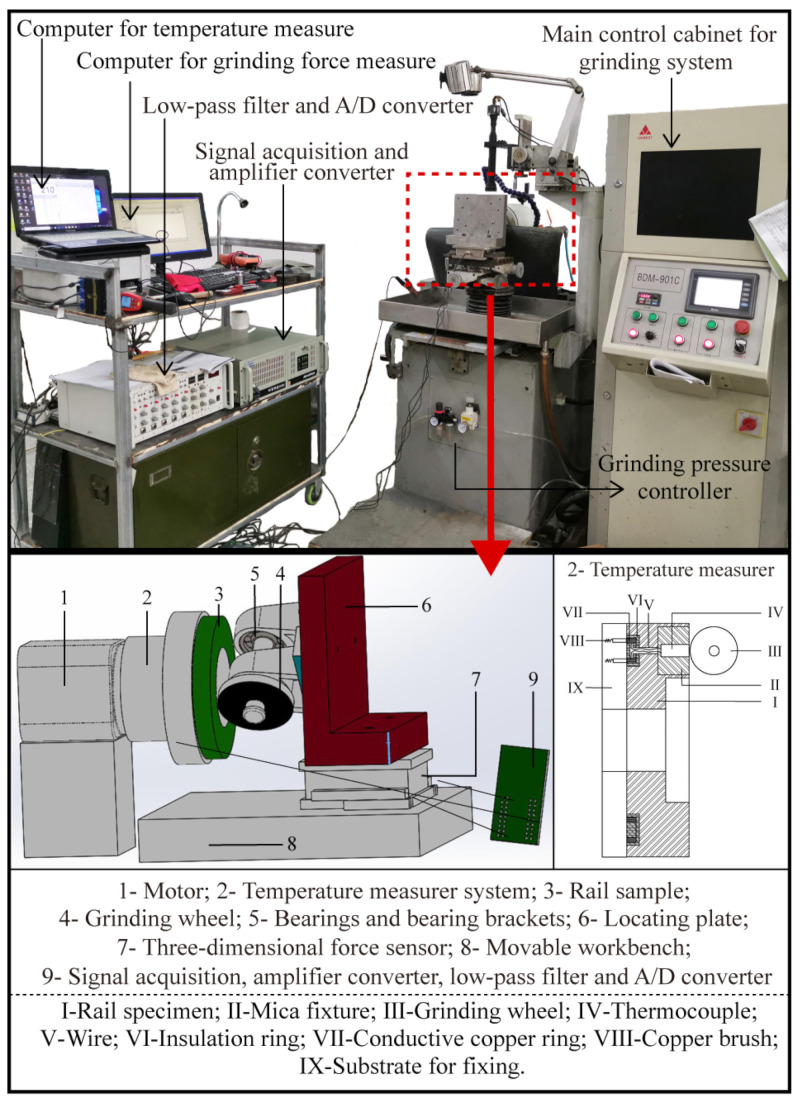
Structure of the grinding test machine.

Research involving high-speed rail grinding is challenging to conduct on rail lines. This is because, first, it is expensive to perform frequent rail grinding tests on in-service railway lines; second, it will result in test errors due to the varying initial state of the railways; and thirdly, the high speed of the rail high-speed grinding train makes it difficult to measure the grinding process and the grinding results. Figure 2 depicts the structure of the self-designed passive grinding test machine utilized for grinding testing in this work. In the preceding study, the structural design, grinding principle, and technical feasibility of the test machine were discussed and explained in detail [16]. Therefore, the content related to the grinding test machine will not be discussed in this study.

Utilizing the grinding force measurement system and the grinding temperature monitoring system of the grinding test machine, the grinding force and grinding temperature were measured during the grinding test. The three-dimensional mechanical sensor was used to measure the grinding pressure and grinding force in the grinding force measurement system. The gathered mechanical signals were analyzed by the Labview force measurement program in the computer via the signal acquisition system and amplifier converter, and the resulting waveform array and oscillogram were constructed in the computer. In the grinding temperature monitoring system, the insertion thermocouple was modified to monitor the grinding temperature, and the thermoelectric effect served as the basis for measuring the temperature. The thermoelectric potential generated by the thermocouple was transmitted to the temperature acquisition module through wires and brushes. Through the A/D converter, low-pass filter, and amplifier, the signals are converted to digital signals, and then they are stored in the computer. The entire test procedures and data processing for the mechanical and thermoelectric signals can also be found in the published study [16], which describes these processes in depth. Equation (1) was used to calculate the rotation speed of rail sample based on the operation speed of the grinding train [16].

(1) $$Rotation\;speed\;of\;rail\;sample\;\left(r/min\right)=\frac{v_{w}\left(km/h\right)\times 10^{6}}{\pi \times d_{s}\times 60}$$

In the formula, *v_w_* is the speed of the grinding train, and *d_s_* is the diameter of the rail sample. Significantly, grinding wheels with varied angles are typically used to trim rails during the high-speed rail grinding process. To control the variables and accurately reflect the influence of grinding time on the high-speed grinding of rails, only the top of the rail material was ground at a vertical angle for testing.

### 2.2. Experimental Materials

**Figure 3:**
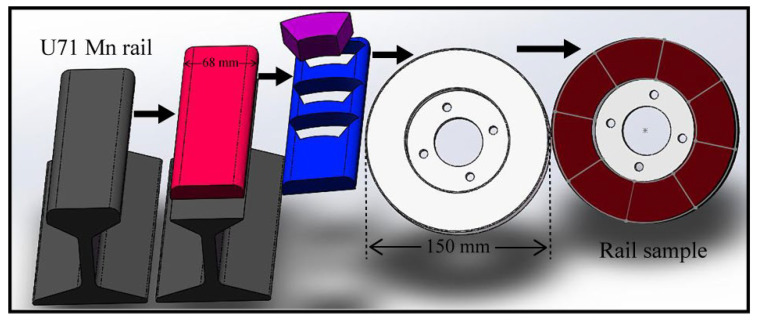
Preparation and morphology of rail sample.

The test rail samples were acquired from the Mn-steel rail installed in the field (Chinese brand: U71Mn). It is widely used on the Chinese railways, and its elemental composition in weight (%) is listed in Table 1. Table 2 provides a summary of the mechanical parameters of this rail steel, and the typical heat treatment for this steel is hot rolling [27]. The rail specimen was assembled directly from the rail head cut into eight identical sectors, as depicted in Figure 3. Before the test, the surface of each rail specimen was machined to the same roughness to ensure similar test conditions.

In this work, a grinding wheel corresponding to the size of the rail sample was used for grinding tests. The material composition of grinding wheels and production formula were developed from the actual high-speed rail grinding wheel [28]. The grinding wheel’s outer diameter, inner diameter, and thickness are 80, 10, and 10 mm, respectively, and its material and composition are detailed in Table 3.

### 2.3. Grinding Test

The grinding tests investigated the effects of passive grinding time and grinding passes on the railway surface. The grinding test settings were based on the actual operational parameters and maintenance requirements of the rail high-speed grinding (HSG-2) train [17]. Each group was subjected to three passive grinding procedures, as detailed in Table 4. The single passive grinding time of Group A was 30 s, whereas that of Group B was 10 s. In Group A and Group B tests, rail samples were ground one, two, and three passes, respectively. The grinding passes represent the total number of repetitions of a single grinding time test. To simulate the forward speed of an 80 km/h high-speed grinding train, the linear velocity of the rail sample was set to 22.2 m per second. The grinding pressure was set at 240 N, which was calculated using the standard pressure load of 12 MPa for passive grinding machines. Additionally, the grinding state of the grinding wheel on the rail is dry grinding, meaning that the auxiliary liquid media material in the grinding process is not involved.

All studies were conducted in the same conditions (20~24 °C, 40~60% relative humidity). Each group of tests was repeated six times with new grinding wheels to acquire the final experimental results. Before the test, the surface of the rail sample and grinding wheels were cleaned with anhydrous ethanol to eliminate the influence of contaminants. Before and after the grinding test, the rail samples and the grinding wheels were weighed using an electronic analytical balance (GL623i; measurement accuracy: 0.001 g). The mass loss of grinding per unit time for the rail sample was defined as the material removal ratio of rail or grinding efficiency, and the grinding ratio was defined as the ratio of grinding mass loss between the rail and grinding wheel. The grinding ratio indicates the grinding life or durability of grinding wheels. The removal of the rail samples was determined by the change in weight. The topography of the surface was observed using an ultra-fine digital microscope (VHX-7000, KEYENCE, Osaka, Japan). The surface roughness of rail samples was measured and recorded by a 3D optical surface profilometer (Ze Gage TM, Zygo, Connecticut, America) before and after the grinding test.

## 3. Results and Discussion

### 3.1. Rail Removal and Grinding Wheel Wear

**Figure 4:**
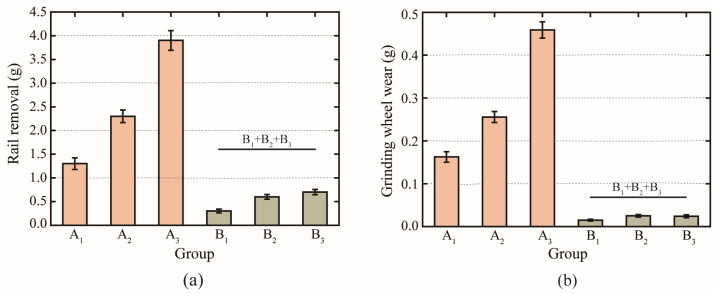
(**a**) Removal of rail samples by grinding wheels and (**b**) wear of grinding wheels at different grinding times.

In order to visualize the effects of grinding time and grinding passes on the grinding results, the weight changes of the rail samples and grinding wheels were measured before and after the grinding test, as seen in Figure 4. With the same number of grinding passes, the longer the single grinding time, the greater the rail removal and grinding wheel loss. Multiple grinding passes of the grinding wheel on the rail samples further increased rail removal and grinding wheel loss. However, under the same total grinding time, the mean value of the sum of rail removal after three passes of 10 s passive grinding (i.e., the sum of rail removal in the B_1_, B_2,_ and B_3_ three groups of grinding tests) was 0.3 g higher than that in the Group A_1_ with only one pass of 30 s passive grinding, while the mean value of the sum of grinding wheel wear loss in the B_1_, B_2_, and B_3_ three groups of grinding tests was 0.1 g lower than that in the Group A_1_. This suggests that the intermittent grinding strategy of extending the grinding time by increasing the grinding passes has effects on the grinding results of passive grinding. The test findings indicate that the short-time and multiple-pass grinding approach is advantageous for increasing rail removal and decreasing grinding wheel wear.

### 3.2. Grinding Efficiency and Grinding Ratio

**Figure 5:**
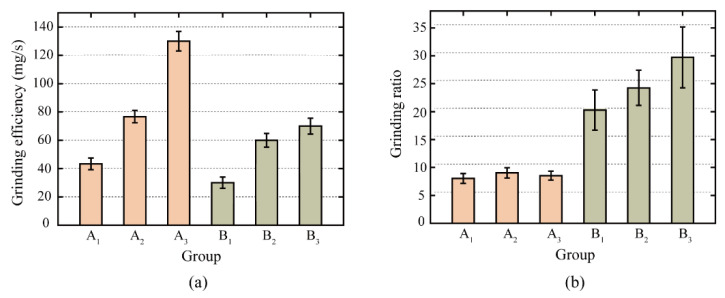
(**a**) Grinding efficiency and (**b**) grinding ratio of grinding wheels at different grinding times.

Figure 5 illustrates the grinding efficiency and grinding ratio for passive grinding tests with various grinding times and passes. For the grinding efficiency, as the number of grinding passes increased, the grinding efficiency of the second pass of repetition was higher than the grinding efficiency of the first pass, and the grinding efficiency of the third pass of repetition was higher than that of the second pass, both for Group A with a single grinding time of 10 s and for Group B with a single grinding time of 30 s. Comparing the Groups A and B with different single grinding times, although the grinding efficiency of the first 10 s passive grinding (B1) was lower than that of the first 30 s passive grinding (A1), the grinding efficiency of the second and third 10 s passive grinding (B2 and B3) was higher than that of the 30 s passive grinding completed in one pass (A1). For a total grinding time of 30 s, the average grinding efficiency was 10 mg/s greater when the passive grinding process was conducted in three times of 10 s than when it was completed in one time of 30 s. In conjunction with the amount of rail removed as seen in Figure 4a, it can be concluded that the passive grinding method with multiple passes helps in enhancing the grinding efficiency.

For the grinding ratio, multi-pass passive grinding with 10 s can improve the grinding ratio of the grinding wheel, whereas multi-pass passive grinding with 30 s cannot considerably improve the grinding ratio of the grinding wheel. This indicates that the short time and multiple passes of passive grinding can effectively extend the service life of high-speed grinding wheels. The passive grinding state of the grinding wheel on the rail was essentially stable when the single grinding time was 30 s. Multiple passes of passive grinding hence have little effect on the durability performance of the grinding wheel.

### 3.3. Morphology and Roughness of Rail Surface

**Figure 6:**
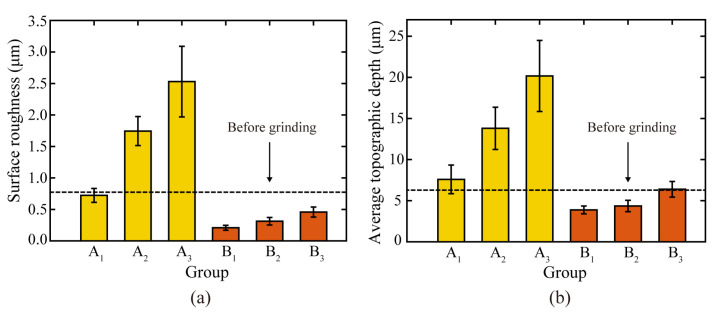
(**a**) Average roughness of rail samples surface and (**b**) average wear depth of grinding marks.

**Figure 7:**
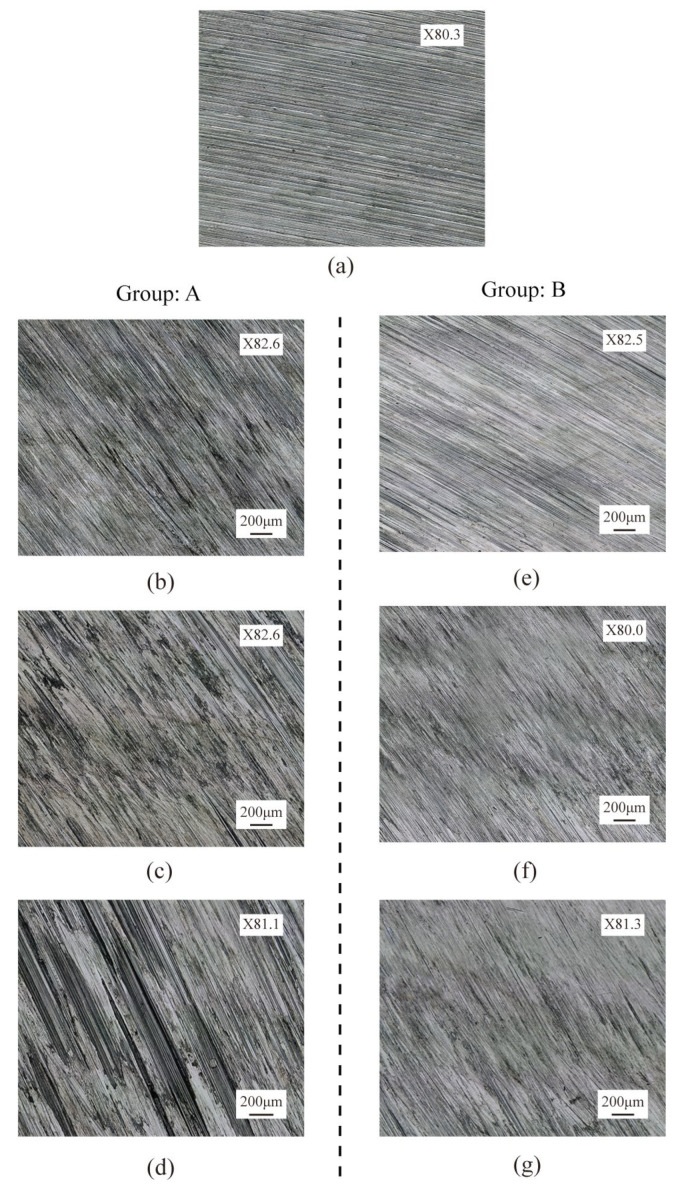
Optical morphologies of rail samples: (**a**) before grinding; (**b**) 30 s first grinding (A_1_); (**c**) 30 s second grinding (A_2_); (**d**) 30 s third grinding (A_3_); (**e**) 10 s first grinding (B_1_); (**f**) 10 s second grinding (B_2_); (**g**) 10 s third grinding (B_3_).

**Figure 8:**
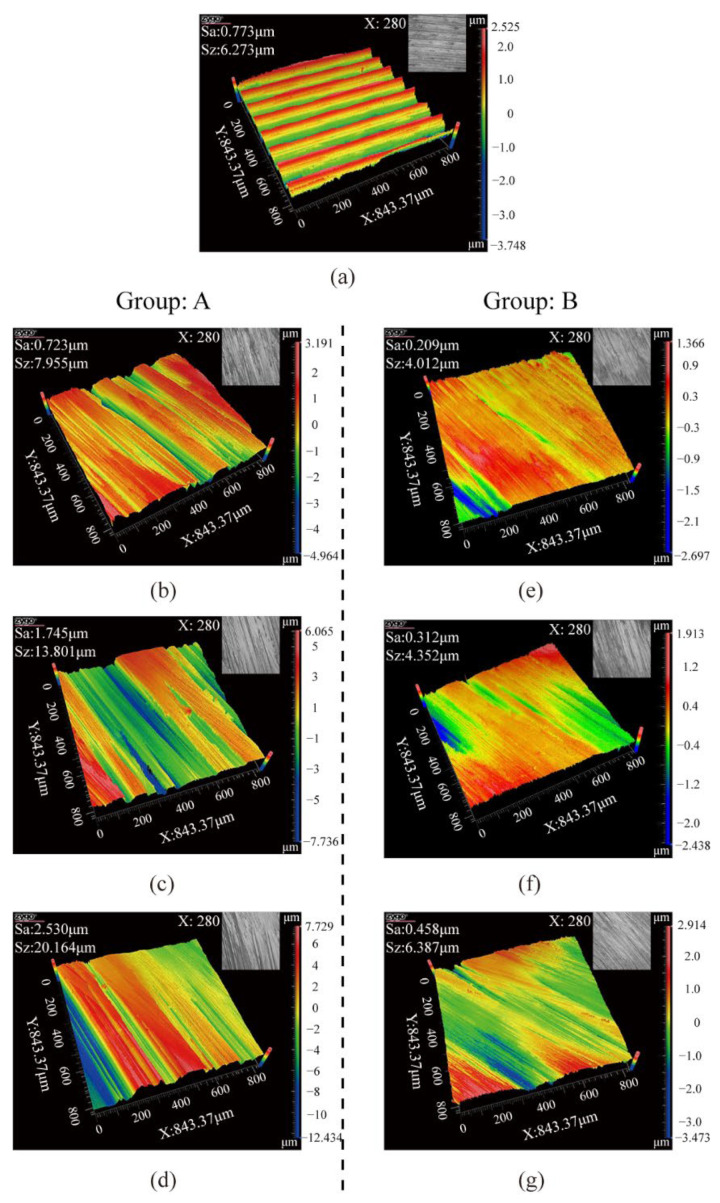
3D profile of rail sample surfaces: (**a**) before grinding; (**b**) 30 s first grinding (A_1_); (**c**) 30 s second grinding (A_2_); (**d**) 30 s third grinding (A_3_); (**e**) 10 s first grinding (B_1_); (**f**) 10 s second grinding (B_2_); (**g**) 10 s third grinding (B_3_).

The surface integrity of rails after grinding is one of the most essential criteria for evaluating grinding quality, as it has a considerable effect on the adhesion characteristics and damage of wheel/rail contact [29]. The surface roughness and grinding marks depth of the rail surface before and after the passive grinding tests were counted, as shown in Figure 6. In addition, the surface morphology and the average state of the three-dimensional profile of the rail samples were visualized, as shown in Figure 7 and Figure 8 respectively. In these figures, Sa and Sz represent the surface roughness and average topographical depth of the rail sample, respectively. After several train rides, rails with good surface roughness from grinding operations would become flat and smooth again [30]. Following a grinding operation, the lower the rail surface roughness and the depth of the grinding marks, the better it is to restore the rail from the grinding profile to the conventional smooth profile. The subsequent use of rail will be harmed if the surface roughness of the rail is excessive or the depth of grinding marks are too deep [31].

Through independent analysis of Groups A and B, it was found that the surface roughness and grinding mark depth of the rail samples rise as the number of grinding passes of the grinding wheel on the rail increases. This indicates that multiple repetitions of passive grinding will continue to change the surface morphology of the rails following passive grinding. A comprehensive comparison of the rail surface grinding results in Group A and Group B reveals that an increase in grinding time will result in an increase in rail surface roughness and grinding mark depth. However, it is noteworthy that differences exist between the surface morphology and surface quality of Group B_3_ rail samples that have been passively ground three times for 10 s and Group A_1_ rail samples that have been passively ground only once for 30 s. Even though the total grinding duration was the same at 30 s, the average surface roughness and average grinding mark depth of the rail samples in Group B_3_ with three grinding passes of 10 s were 0.46 µm and 6.39 µm, respectively, while they were 0.72 µm and 7.96 µm in Group A_1_ with one grinding pass of 30 s. The surface roughness and average grinding mark depth of the rail samples in Group B_3_ were lower than those in Group A_1_, indicating that the short time and multiple passes grinding strategy improved the grinding quality.

### 3.4. Surface Topography of Grinding Wheel

**Figure 9:**
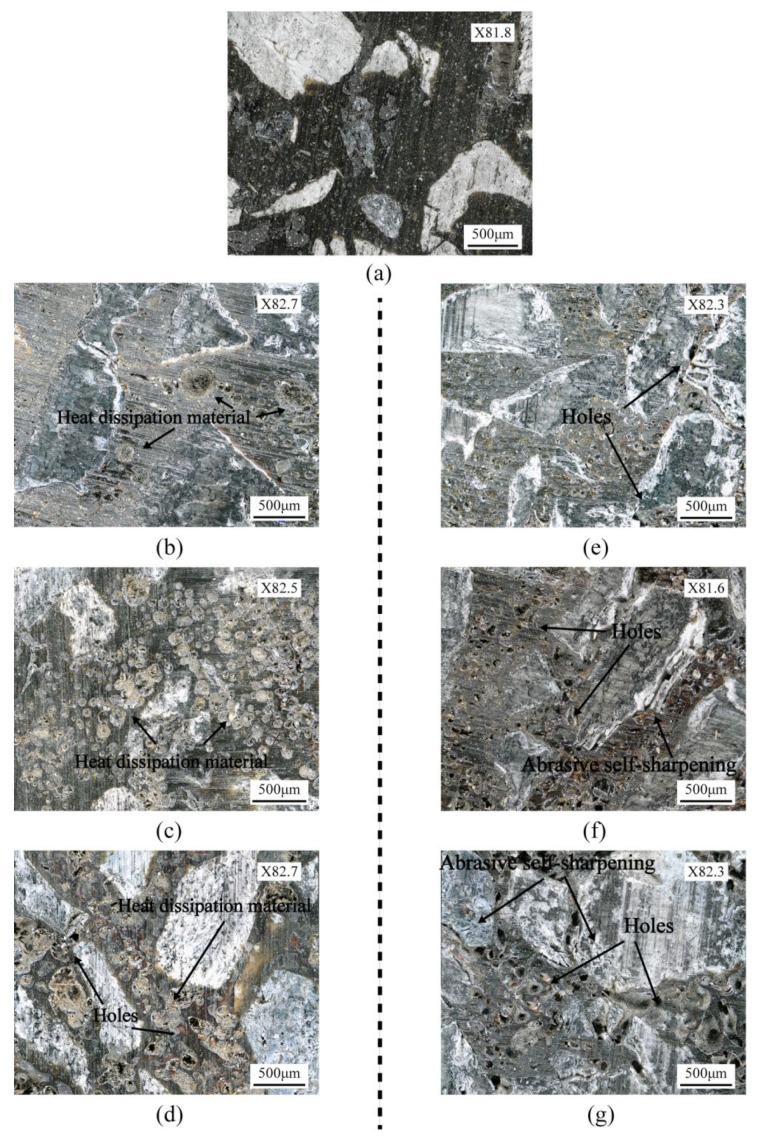
The surface of grinding wheel samples: (**a**) before grinding; (**b**) after the first pass of 10 s passive grinding (B_1_); (**c**) after the second pass of 10 s passive grinding (B_2_); (**d**) after the third pass of 10 s passive grinding (B_3_); (**e**) after the first pass of 30 s passive grinding (A_1_); (**f**) after the second pass of 30 s passive grinding (A_2_); (**g**) after the third pass of 30 s passive grinding (A_3_).

Figure 9 depicts the surface morphology of the grinding wheel before and after the grinding tests. The surface of the grinding wheel exhibited varying degrees of wear as grinding time and grinding passes increased, and the self-sharpening state of grinding wheels differed as well. Under the conditions of a single grinding time of 10 s, the grinding wheel surface gradually displayed slight wear as the number of grinding passes increased; under the conditions of a single grinding time of 30 s, the wear on the grinding wheel surface tended to become pronounced as the number of grinding passes increased. After 30 s of passive grinding with three passes, visible hole structures emerged on the surface of the grinding wheel, as well as slight wear and self-sharpening of the zirconia abrasive. With the same total grinding time, the wear and self-sharpening degree of the abrasives on grinding wheel surfaces after three 10 s passive grinding passes were comparable to those after one 30 s passive grinding pass; however, the state of the resin binder and the heat dissipation filler (Pyrite: main component FeS_2_) of grinding wheels was significantly different. The filler pyrite was held by the resin binder and dispersed among the abrasives. In the 10 s passive grinding of Group B, the pyrite filler (FeS_2_) in the grinding wheel was depleted gradually as the number of grinding passes increased, and the loss of pyrite in the grinding wheel after three passes of 10 s passive grinding was significantly greater than that after a single pass of 30 s passive grinding. In contrast, only a small amount of pyrite filler was lost in the grinding wheel during the first 30 s of passive grinding for Group A. The pyrite was gradually depleted as the number of 30 s passive grinding passes increased, and due to the disintegration of the oxidation process, holes appeared in the structure of the grinding wheel after the second and third passive grinding passes. These holes will increase the wear and cracking of the phenolic resin binder during the grinding process, resulting in a bigger hole structure on the grinding wheel surface, which in turn accelerates the wear and self-sharpening of the grinding wheel.

Pyrite (*FeS*_2_) is frequently used as a filler in the manufacturing of high-speed rail grinding wheels. In order to minimize rail oxidation and burning caused by grinding, one of its primary roles is to reduce the transiently high temperatures generated during the grinding operation. Pyrite absorbs grinding heat and combines with oxygen to break down into ferric oxide (*Fe*_2_*O*_3_) and sulfur dioxide (*SO*_2_), as shown in Equation (2).

(2) $$4FeS_{2}+11O_{2}\overset{grinding\;heat}{\to }2Fe_{2}O_{3}+8SO_{2}$$

The surface morphology of the grinding wheel after grinding indicates that the phase in which pyrite exerts its heat absorption effect is primarily concentrated during the initial phase of passive grinding. Since the dry grinding process without grinding fluid has just begun at this stage, a substantial amount of grinding heat will be produced, resulting in a high immediate temperature. The pyrite will then absorb the grinding heat and react with oxygen to lower the grinding temperature. As the grinding process continues, the vast amount of grinding debris formed in the grinding process will absorb a portion of the grinding heat, while the grinding heat will progressively conduct and diffuse to the outside world via media such as the rail, grinding wheel, and air. This causes the grinding heat to begin to gradually accumulate, resulting in a steady increase in grinding temperature, as opposed to continuing to produce a large amount of sudden high temperature. This change will moderately delay the oxidation reaction rate of pyrites, resulting in the pyrite’s progressive depletion when the grinding temperature is kept increasing.

### 3.5. Grinding Force

**Figure 10:**
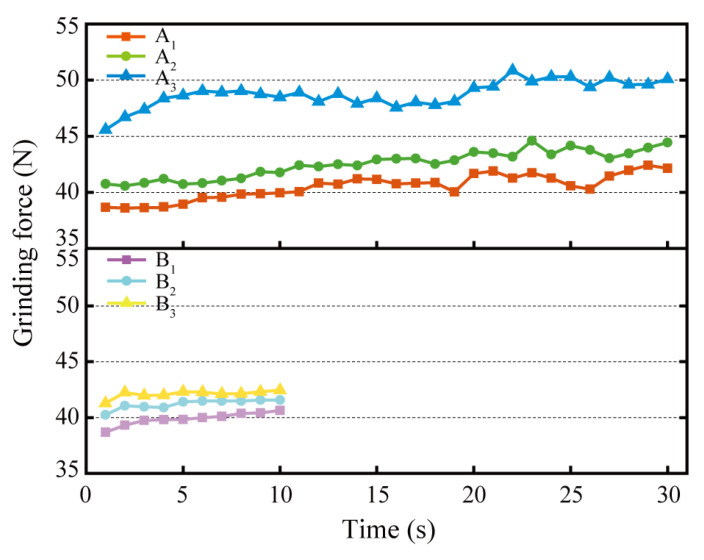
Grinding forces of grinding wheels on rail samples under different grinding times.

The grinding forces of the grinding wheels on the rail samples in each test group are shown in Figure 10. According to the figure, the grinding force rose as the number of grinding passes increased for both a single grinding time of 10 s and a single grinding time of 30 s. Contrasting the grinding forces of Group A_1_ and Group B for the same total grinding time of 30 s reveals that the average grinding forces of Groups B_1_, B_2_, and B_3_ were marginally higher than those of Group A_1_ in the first 10 s, middle 10 s, and final 10 s of the grinding process, respectively. Moreover, the change trend of grinding force was more stable in Group B than in Group A_1_. These reasons make the grinding effect of rail samples by high-speed grinding wheels in Group B better than in Group A_1_, and explain why the intermittent and short-time passive grinding process can make the grinding process of the grinding wheel on the rail more stable. In addition, the grinding forces of A_2_ and A_3_ in Group A fluctuated, particularly in the second half of the grinding time exceeding 15 s. Comparing the three 30 s passive grinding tests in Group A (A_1_, A_2_, and A_3_), the grinding force in Group A_2_ increased slightly relative to Group A_1_, whereas the grinding force in Group A_3_ increased significantly relative to Group A_2_. For these reasons, the comprehensive performance of the passive grinding force in three passes of 10 s grinding time was better than that after one pass of 30 s. Not only was the maximum value of the grinding force (43.39 N) in three passes of 10 s higher than the maximum value of the grinding force (42.41 N) in one pass of 30 s, but also the stability of the grinding force was better. The maximum value of the grinding force in the third pass of 30 s grinding time was 50.35 N, which was greater than the value of 43.99 N in the second pass of 30 s grinding time and the value of 42.41 N in the first pass of 30 s grinding time. Nevertheless, its stability was weaker, which could negatively impact the rail grinding results.

In general, the grinding force of high-speed grinding wheels on rails in Groups A_1_, A_2_, and A_3_ exhibited an upward trend as the number of grinding passes and cumulative grinding time increased. When the grinding wheel produces more and deeper grinding marks on the rail surface, the average grinding area of the abrasive decreases during passive grinding. Therefore, more energy is required to cut off a unit volume of rail material, which is macroscopically presented as an increase in grinding force. This relates to the dimensional effect of the grinding force of the grinding wheel. In addition, the surface state of the rail in 3.3 shows that the surface roughness and the depth of grinding marks of the rail exhibit a trend of first decreasing and then increasing with the increase of grinding passes and the accumulation of grinding time. This indicates that the grinding force generated by the grinding wheel on the rail during passive grinding is proportional to the rail surface roughness after surface defects are removed, namely, the higher the rail surface roughness, the greater the grinding force generated by the grinding wheel on the rail during passive grinding.

### 3.6. Grinding Temperature

**Figure 11:**
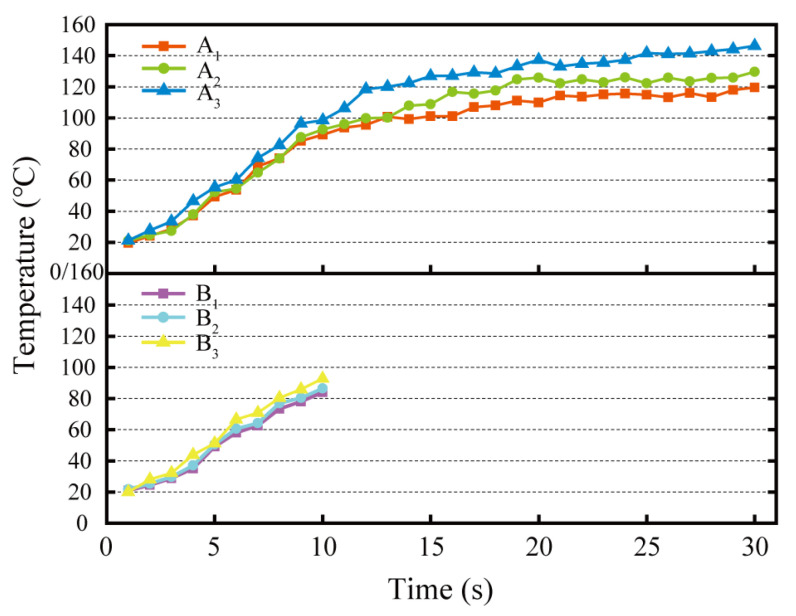
Grinding temperature of rail samples by grinding wheels under different grinding times.

Figure 11 illustrates the grinding temperatures of the grinding wheel on the rail samples under varied grinding time conditions. As can be seen, there are also variations in the grinding temperatures for different grinding times and grinding passes.

Grinding time is one of the influential factors on rail grinding temperature. The longer the grinding time, the more the grinding heat accumulates, resulting in a higher grinding temperature. In addition, as the number of grinding passes increases, the grinding temperature rises faster and is higher at the same point in time. At the end of the third pass of 30 s grinding, the highest grinding temperature of 148.4 °C in the six groups of grinding tests was exhibited. The variation of rail grinding temperature with time for rail grinding wheels is related to the energy consumed to remove a unit volume of metal, i.e., the specific grinding energy (*E_s_*), as shown in Equation (3) [32].

(3) $$E_{s}=\frac{v_{s}F_{t}}{v_{w}a_{p}b}$$

In the formula, vs. is the linear velocity of grinding wheel (m/s), *F_t_* is the grinding force (N), *v_w_* is the linear velocity of rail sample (m/s), *a_p_* is the grinding depth, and *b* is the grinding width. The higher the grinding force, the greater the specific grinding energy of passive grinding, which generates more grinding heat and accelerates the rate at which the grinding temperature rises. Even though the grinding temperature increased with grinding time and the number of grinding passes, the maximum grinding temperature did not exceed 400 °C in any of the grinding test groups, which was below the oxidation burn temperature of the rail material [24]. As seen in Figure 7, there is no “blue” or “yellow” rail oxidation burn exhibited on the ground of rail samples. Burning of the rail surface during the active rail grinding process is common [16]. Monitoring results of grinding temperature demonstrate that the passive high-speed rail grinding procedure is less likely to cause rail burn due to grinding maintenance than the active rail grinding process.

### 3.7. Comprehensive Evaluation and Analysis

According to the high-speed grinding conditions of rails, three passes of passive grinding tests with 30 s grinding time in Group A and 10 s grinding time in Group B were designed to investigate the effects of a single grinding time and the number of grinding passes on grinding wheel performance and passive grinding results. As stated in the introduction, the essence of increasing the number of passive grinding passes is to extend the grinding time of the grinding wheel on the rails in an alternative manner. Nevertheless, based on the results of the grinding tests, even if the total grinding time is the same, the grinding performance of the grinding wheels and the passive grinding results of the rail in multiple grinding passes are relatively distinct from those in a single grinding pass.

Independent analysis of the Group A grinding test results revealed that the grinding efficiency, rail removal in a single pass, grinding temperature, surface roughness of the rail samples after the grinding tests, and depth of the grinding marks increased as the number of rail grinding passes increased, even though the other grinding parameters such as grinding time, grinding pressure, and grinding speed remained unchanged. This is related to the grinding state of the grinding wheel on the rails. Insufficient grinding time prevents the passive grinding behavior of grinding wheels on the rail from reaching a stable peak condition. In the three passes of 30 s of passive grinding, the grinding ratio of the grinding wheel stabilized, but the surface roughness of the rail samples and the grinding force increased with each additional pass. This change can be regarded as a continuation of the grinding state taking over the rail grinding again after the last time rail passive grinding. The continuity of the values for grinding force and rail surface roughness across various grinding passes demonstrates this point. And the grinding test results indicated that the passive grinding for 3 passes of 30 s improved the grinding efficiency without affecting the service life of the grinding wheels, which is advantageous for increasing the amount of rail grinding and can be used for grinding maintenance of the rail surface with severe damage.

Comparing the grinding test results of Group A_1_ with those of Groups B_1_, B_2_, and B_3_, it was discovered that, under the same conditions of total grinding time and remaining grinding parameters, the grinding performance of the grinding wheels and the grinding results of the rail samples were slightly different when the 30 s of passive grinding was completed in one pass versus when it was completed in three passes of 10 s. Not only were the surface roughness and grinding mark depth of the rail sample after the third 10 s of passive grinding lower than those after the first 30 s of passive grinding, but the total grinding removal of the rail after the three 10 s passive grinding passes was slightly greater than that after the first 30 s of passive grinding, and the total grinding wheel wear was also lower after the third 10 s of passive grinding pass. Equation (4) displays the empirical formula for grinding efficiency per unit time [33].

(4) $$Q_{w}=\frac{v_{s}\cdot \sqrt[{x}]{F_{t}}}{f}$$

In the formula, *Q_w_* is the grinding efficiency per unit time (mg/s), vs. is the linear velocity of grinding wheel (m/s), *F_t_* is the grinding force (N), and *x* and *f* are the grinding constants. According to Figure 10, the grinding forces of Groups B_1_, B_2_, and B_3_ are somewhat higher than those of Group A_1_ at the corresponding grinding time node. Consequently, Equation (4) demonstrates that the grinding efficiency and total rail removal of multi-pass passive grinding are higher than those of single-pass passive grinding. In addition, the grinding forces of Group A_1_ in the final 10 s of grinding fluctuated significantly, while the grinding forces of Groups B_1_, B2, and B3 in the three passes of 10 s grinding tests were relatively stable. This indicates that the short-time state of passive grinding is more steady. Furthermore, the monitoring results of the grinding temperature revealed that although the maximum grinding temperature increased after each 10 s of passive grinding, the grinding temperature after 30 s of passive grinding was higher than that of all 10 s of passive grinding tests. This is due to the accumulation of grinding heat caused by the continuous grinding on the one hand, and the filler (Pyrite/FeS_2_) of the rail grinding wheel on the other hand. The accumulation of grinding heat caused by prolonged dry grinding accelerates the wear of the grinding wheels. This would not only shorten the service life of the grinding wheel but also limit its grinding performance, thus diminishing the grinding quality of the wheel on the rail [28,34]. For the implementation of preventive grinding of rails, a short-time grinding program of 1 or 3 passes is reasonable because the low degree of rail disease does not require a large amount of rail grinding.

In conclusion, the passive grinding operation with the short time and multiple passes is conducive to the performance of the passive grinding, improving the grinding efficiency of the rail within the same grinding time, optimizing the grinding quality of the grinding wheel to the rail, and extending the service life of the grinding wheel.

## 4. Conclusions

This study investigated the effects of grinding time and number of grinding passes on the grinding efficiency, rail grinding effect, and service life of grinding wheels of the passive grinding process, as well as their underlying principles, under the conditions of a constant relative rail motion speed and grinding wheel deflection angle. The main conclusions are as follows:

1. The passive rail grinding strategy of repetitions with three passes is conducive to overcoming the challenge that the grinding removal and rail surface roughness cannot reach the desired level during the high-speed rail grinding process to achieve the expected rail grinding objective.
2. When the total grinding time was thirty seconds, the grinding removal and rail surface roughness of the passive grinding performed in three passes of ten seconds were better than those of the passive grinding completed in one pass of thirty seconds. It indicates that the passive grinding mode with short time and multiple passes improves the grinding efficiency of the high-speed rail grinding process and the rail grinding quality compared to completing the rail grinding operation in a single pass.
3. The maximum grinding temperature at the conclusion of the testing was 148.4 °C, which is lower than the initial temperature of 400 °C at which the rail produces oxidation burns. Due to the fast relative motion speed of grinding wheels and rails, high-speed grinding of rails does not easily cause apparent oxidation burns on the rail surface.
4. Three passes of intermittent grinding for ten seconds provide a greater grinding ratio, lower grinding temperature, and more steady grinding force than one pass of single grinding for thirty seconds. It reveals that the short-time and multiple-pass passive grinding mode can reduce the accumulation of grinding heat, slow the wear of the grinding wheel to extend its service life, and stabilize the grinding process of the grinding wheel on the rail.

## Figures and Tables

**Table 1:** Chemical compositions (wt%) of U71Mn rail steel [27].

Elemental	C	Si	Mn	P	S	V	Nb
content	0.65–0.76	0.15–0.58	0.70–1.20	≤ 0.035	≤ 0.030	0.030	≤0.010

**Table 2:** Mechanical properties of U71Mn rail steel [27].

Steel Material	Tensile Strength σb (MPa)	Elongation Rate δ (%)	Fracture Toughness K_IC_ (Mpa·m^1/2^)	Hardness (HB)
U71Mn	≥880	≥ 10	≥26	260–300

**Table 3:** Composition of passive grinding wheels.

Grinding Wheel Type	Hot Pressed Resin Grinding Wheel
Abrasive	fused alumina zirconia, brown fused alumina, ceramics corundum abrasives
Abrasive size	14–60 mesh
Binder	high toughness heat-resistant resin
Auxiliary materials (fillers/heat dissipation material)	pyrites (FeS_2_)

**Table 4:** Grinding parameters of grinding time on grinding tests.

No.	Grinding Wheel Angle	Rotation Speed of Rail Sample	Grinding Pressure	Grinding Time	Grinding Passes
A_1_	45°	2831 r/min	240 N	30 s	1
A_2_	45°	2831 r/min	240 N	30 s	2
A_3_	45°	2831 r/min	240 N	30 s	3
B_1_	45°	2831 r/min	240 N	10 s	1
B_2_	45°	2831 r/min	240 N	10 s	2
B_3_	45°	2831 r/min	240 N	10 s	3

## Data Availability

The datasets used or analyzed during the current study are available from the corresponding author on reasonable request.
